# PD-L1 Expression Varies in Thyroid Cancer Types and Is Associated with Decreased Progression Free Survival (PFS) in Patients with Anaplastic Thyroid Cancer

**DOI:** 10.3390/cancers16213632

**Published:** 2024-10-28

**Authors:** Leila Shobab, Deema Al-Souri, Liza Mathews-Kim, Matthew McCoy, William Kuenstner, Gretchen K. Hubbard, Sonam Kumari, Jiling Chou, Wen Lee, Jennifer Rosen, Joanna Klubo-Gwiezdzinska, Michael Atkins, Leonard Wartofsky, Vasyl Vasko, Kenneth Burman

**Affiliations:** 1 Department of Medicine, Division of Endocrinology, MedStar Washington Hospital Center, Washington, DC 20010, USA; deema.alsouri@hotmail.com (D.A.-S.); kenneth.d.burman@medstar.net (K.B.); 2 School of Medicine, Georgetown University, Washington, DC 20057, USA; lrm107@georgetown.edu; 3 Innovation Center for Biomedical Informatics, Georgetown University Medical Center, Washington, DC 20007, USA; 4 CARIS Life Sciences, Phoenix, AZ 85040, USA; ghubbard@carisls.com; 5 National Institute of Diabetes and Digestive and Kidney Diseases (NIDDK), National Institutes of Health (NIH), Bethesda, MD 20892, USA; 6 MedStar Health Research Institute, Columbia, MD 21044, USA; 7 Department of Pathology, MedStar Washington Hospital Center, Washington, DC 20010, USA; 8 Department of Surgery, MedStar Washington Hospital Center, Washington, DC 20010, USA; jennifer.e.rosen@medstar.net; 9 Division of Hematology/Oncology, MedStar Georgetown University Hospital, Washington, DC 20007, USA; mba41@georgetown.edu; 10 Department of Pediatrics, Uniformed Services University of the Health Sciences, Bethesda, MD 20814, USA; vasyl.vasko@gmail.com

**Keywords:** thyroid cancer, radioactive iodine refractory, immunotherapy for thyroid cancer, PD-L1 expression in thyroid cancer, thyroid cancer tumor microenvironment

## Abstract

Background: Thyroid cancer (TC) remains a significant clinical challenge worldwide, with a subset of patients facing aggressive disease progression and therapeutic resistance. Immune checkpoint inhibitors targeting programmed death-ligand 1 (PD-L1) have emerged as promising therapeutic approaches for various malignancies, yet their efficacy in TC remains uncertain. The objective of this study was to investigate PD-L1 expression in aggressive TC and its association with histological subtypes, molecular mutation, and progression-free survival. Methods: This is a retrospective study of patients with advanced TC seen in two tertiary health care centers. Included in this study were patients with advanced TC with recurrence or progression on therapy for whom tumor molecular profiling and PD-L1 status were available. Kaplan–Meier estimators were utilized to analyze the progression-free survival (PFS) between patients with PD-L1 positive and negative status in Anaplastic TC (ATC) subgroup. Results: A total of 176 patients with advanced thyroid cancer were included (48.9% female). Of the patients, 13 had ATC, 11 Medullary TC (MTC), 81 Papillary TC Classic Variant (PTCCV), 20 Follicular TC (FTC), 8 Oncocytic TC (OTC), 10 Poorly Differentiated TC (PDTC), and 30 had the Papillary TC Follicular Variant (PTCFV). *BRAF* mutation was present in 41%, *TERT* in 30%, *RAS* in 19%, *TP53* in 10%, and *RET* in 8.6% of patients. PD-L1 positivity was significantly different across different TC types and histological subtypes (*p* < 0.01): Patients with OTC had the highest frequency of PD-L1 positivity (71%), followed by ATC (69%), PTCCV (28.5%), and FTC (11%). Patients with MTC and PTCFV did not exhibit any PD-L1 positivity. *TP53* mutation was positively associated with PD-L1 expression (21.6% vs. 7.5%, *p* = 0.03), and *RAS* mutation was negatively associated with PD-L1 expression (8.1% vs. 24.2% *p* = 0.04). Among patients with ATC, positive PD-L1 expression was associated with lower PFS (*p* = 0.002). Conclusions: PD-L1 expression varies across different TC types and histological subtypes and may be modulated by the mutational landscape. PD-L1 expression in ATC is associated with shorter PFS. Follow up studies are warranted to elucidate the molecular mechanism driving the observed differences in immune pathways, potentially paving the way for the development of more effective and personalized immune therapies for patients with aggressive TC.

## Simple Summary

Our study examined PD-L1 expression in advanced Thyroid Cancer (TC) and its relationship with histological subtypes, molecular mutations, and progression-free survival (PFS). Analyzing data from 176 patients with advanced TC, our study found significant variability in PD-L1 expression with Oncocytic Thyroid Cancer (OTC) and Anaplastic Thyroid Cancer (ATC) exhibiting the highest frequencies of PD-L1 expression, while Medullary TC (MTC) and Papillary TC Follicular Variant (PTCFV) did not show any PD-L1 expression. Notably, PD-L1 positivity correlated with shorter progression-free survival (PFS) in ATC and was associated with *TP53* mutation. These findings suggest that PD-L1 expression, combined with genetic profiling could inform personalized immunotherapy strategies for aggressive forms of TC, emphasizing the need for further research to validate these biomarkers and enhance treatment efficacy.

## 1. Introduction

Thyroid cancer (TC) represents the most prevalent endocrine neoplasm worldwide, with increasing incidence and mortality rates [1]. TC encompasses a spectrum of heterogenous types, including Differentiated (DTC), Anaplastic (ATC), and Medullary (MTC) thyroid cancer. Among these, DTC is the most common type accounting for over 85% of cases [2]. Differentiated thyroid cancer is further categorized into Papillary Thyroid Cancer (PTC, approximately 70–80% of DTC), Follicular Thyroid Cancer (FTC, 10%), Oncocytic Thyroid Cancer (OTC, 2–4%), and Poorly Differentiated TC (PDTC, 5–10%) [3,4]. Papillary TC is further subdivided into the Classic Variant (PTCCV) and Follicular Variant (PTCFV). Undifferentiated TC may give rise to ATC, which has a dismal prognosis, characterized by a median survival of 5–6 months and a 1-year survival rate of approximately 20% [5]. MTC is notable for its significant mortality rates, contributing to 9% of TC-related deaths although it represents only 2–3% of all TCs [6]. In patients with DTC, despite a 5-year survival that exceeds 95%, 15–20% may progress to locoregional or distant metastasis, with about a third becoming refractory to conventional therapy with radioactive iodine (RAI). These patients exhibit a markedly reduced 10-year survival rate of only 10% [4]. Currently available systemic therapies, including tyrosine–kinase inhibitors (TKIs) show variable efficacy and significant toxicity, but have yet to demonstrate clear overall survival benefits [7,8,9]. Therefore, there is an urgent need to better understand the molecular mechanisms underlying aggressive, treatment-resistant forms of TC, in order to develop more efficacious and targeted therapies. 

In recent years, there has been a paradigm shift in cancer treatment with the advent of immune-based therapies, particularly immune checkpoint blockade (ICB). Immune checkpoint molecules are expressed on various immune cells, such as activated T cells, macrophages and dendritic cells (DCs) and regulate immune activation and prevent excessive immune responses that could lead to autoimmune disorders [10]. In cancer, tumors often exploit these inhibitory checkpoints to create an immunosuppressive environment that enables immune evasion. Programmed death 1 (PD-1), an immune checkpoint receptor predominantly expressed on T cells, interacts with its ligand, PD-L1 to promote immune tolerance and regulate inflammation [11]. PD-L1 expression represents a pre-requisite for the use of immune checkpoint inhibitors in cancer treatment in some but not all cancers. Inhibition of immune checkpoint proteins, including PD-1, PD-L1, CTLA-4 represent a significant breakthrough in the cancer immunotherapy, showing notable success in treating patients with melanoma, renal cell carcinoma, head and neck cancers or non-small-cell lung cancer [12,13,14,15].

Historically, TC has been categorized as an ‘immunologically cold’ tumor, suggesting limited potential for successful application of immune-based treatments [16,17]. This classification was primarily based on the analyses of The Cancer Genome Atlas (TCGA) data, which predominantly included low- to intermediate-risk DTC cases associated with a low tumor mutation burden (TMB) and low PD-L1 expression [18,19]. However, recent studies on advanced TC, which have also included ATC and PDTC, suggest that the immune system plays a critical role in TC pathogenesis and progression. Emerging literature describes a complex immune response within the tumor microenvironment (TME) of TC, influenced by driver mutations, cancer types and histological subtypes [20,21,22,23]. Recent transcriptomic analyses have revealed a dynamic interplay between the immune system and TC progression, from benign thyroid tissue to differentiated and ultimately dedifferentiated or anaplastic forms [24,25]. These studies highlight distinct immune response patterns within the TME, starting with a stress-responsive metabolic deregulatory state in early disease stages, evolving into heightened inflammatory pathways, and eventually shifting to a defective mitotic state and a mesenchymal/fibrotic phenotype in advanced stages [24]. Despite these advances, significant gaps remain in our understanding of immune responses across different TC types and histological subtypes. Existing literature reports broad variability in PD-L1 expression in TC, ranging from 5 to 87%, which may be due to differences in population tested, detection methods, assay techniques, and interpretation [26,27,28]. This variability underscores the need for further investigation into the pathophysiological basis of PD-L1 expression heterogeneity in TC and its potential relationship with disease outcome. 

The objective of our study was to measure PD-L1 expression across different TC types and histological subtypes of DTC and evaluate its association with mutational status and Progression Free Survival (PFS) in patients with advanced TC. 

## 2. Methodology/Study Design

This is a retrospective study of patients with advanced thyroid cancer who were followed at the MedStar Washington Hospital Center and MedStar Georgetown University Hospital between January 2010 and April 2024. The study was approved by the Institutional Review Board at MedStar Health Research Institute/Georgetown University, ensuring adherence to ethical standards and patient confidentiality. Eligible patients were included in this study who had advanced TC (Stage II in age <45 before and <55 after 2018 and Stage 3 and 4 in age ≥ 45 before 2018 and ≥55 after 2018) with recurrence or progression on therapy for whom tumor molecular profiling was available. Patients were excluded if they were under 18 years of age or if data on molecular mutation testing were unavailable. Clinical, pathological, and treatment responses data were collected from patient charts. 

**Molecular Mutational Analysis:** The molecular mutational studies were performed by Caris Life Sciences according to their standard protocol. In brief, formalin-fixed paraffin-embedded (FFPE) tissue samples from thyroid carcinoma patients treated at MedStar Hospital Center or MedStar Georgetown University Hospital were analyzed by a CLIA/CAP-certified lab (Caris Life Sciences, Phoenix, AZ, USA). Analyses performed include next-generation sequencing (NGS), whole-transcriptome sequencing (WTS), and immunohistochemistry (IHC) for molecular and genomic features including tumor mutational burden (TMB), tumor mutations and fusions, microsatellite instability (MSI), PD-L1 positivity, and mismatch repair deficiency (MMRd). 

**DNA Next-Generation Sequencing:** Next-generation sequencing (NextSeq or NovaSeq 6000, Illumina, Inc., San Diego, CA, USA) was performed on genomic DNA using a 592-gene panel or whole-exome sequencing (700 genes at high coverage and read depth), with genetic variant calling by board-certified molecular geneticists as previously described [29]. TMB, genomic loss of heterozygosity (gLOH), and MSI were calculated and called as previously described [30].

**PD-L1 Immunohistochemistry:** For PD-L1 status, immunohistochemistry (IHC) was performed on FFPE sections of glass slides. The slides were stained using automated staining techniques, per the manufacturer’s instructions, and were optimized and validated per Clinical Laboratory Improvement Amendments (CLIA)/CAO and ISO requirements. A board-certified pathologist evaluated all IHC results independently. The primary antibody used against PD-L1 was SP142 (Spring Biosciences, San Francisco, CA, USA). The staining was regarded as positive if its intensity on the membrane of the tumor cells was ≥2+ (on a semi quantitative scale of 0–3: 0 for no staining, 1+ for weak staining, 2+ for moderate staining, or 3 + for strong staining) and the percentage of positively stained cells was ≥5%.

## 3. Statistical Analysis

Descriptive statistics were used to summarize demographic characteristics, clinical and pathological presentations, and molecular mutations across the study population, as well as across groups defined by gender, histological subtypes, and PD-L1 expression. Categorical variables were summarized using frequencies and percentages, while continuous variables were reported as means with standard deviations (SDs) for normally distributed data or medians with interquartile ranges (IQRs) for non-normally distributed data.

To assess group differences, Fisher’s exact test or the chi-squared test were applied for categorical variables, and the Kruskal–Wallis test was used for comparing non-normally distributed continuous variables. The normality of continuous variables was evaluated using the D’Agostino–Pearson test.

The multivariate cox proportional hazard model was used to evaluate how PD-L1 is associated with PFS-controlling covariates. Covariates were selected based on a bivariate analysis using PD-L1 and the elastic-net penalized cox proportional hazard model. An adjusted hazard ratio (HR) was estimated, and the 95% confidence interval (CI) was calculated using the Wald method. The results showed no significant difference in progression-free survival (PFS) between the PD-L1-positive and PD-L1-negative groups (1.18 95% CI (0.76, 1.81), *p* = 0.464) after adjusting for histopathology type, RAS mutation, TERT mutation, TP53 mutation, bone metastases, and age. To investigate the association between PD-L1 expression and survival outcomes in the ATC subgroup, Kaplan–Meier survival curves were generated, and comparisons between groups were made using the log-rank test. Progression-free survival (PFS) and overall survival (OS) were the primary outcomes analyzed. Hazard ratios (HRs) with corresponding 95% confidence intervals (CIs) were reported. Kaplan–Meier (KM) estimates and a multivariate Cox proportional hazards model were used to assess the effects of TP53 and BRAF mutations and their interactions with PD-L1. All statistical tests were two-sided, and a *p*-value of less than 0.05 was considered statistically significant. Data analysis and visualizations, including the creation of survival curves, were performed using R software 4.3.1 and Python 3.12.4. 

## 4. Results 

### 4.1. Clinical and Pathological Presentation

A total of 176 patients were included in this study. (Table 1) There were 86 (49%) female and 89 (51%) male patients. The median age at diagnosis was 58 years in females and 60 years in males (58.0 [43.0, 69.0], 60.0 [50.0, 69.0] *p* = 0.179). The majority of patients were white (60%) followed by African American (25%), Asian (3.6%), Latino (0.6%), and other races (10.8%). There were 13 patients with ATC (7%), 11 with MTC (6%), 81 (46%) with PTCCV, 20 with FTC (11%), 10 with PDTC (6%), 30 with PTCFV (17%), and 8 with (OTC, 4.5%). The clinicopathological presentation within each histological subtype is presented in Table 1. Patients with PDTC had the largest tumors (median 7.0 [6.0, 7.5]); overall, 46% of patients had positive surgical margins, with the highest prevalence in the ATC (100%) and PDTC (57%) patients. Extrathyroidal extension was present in 43% of the overall cohort with the highest prevalence in the ATC patients (100%) followed by the PDT patients (71%). Distant metastasis was present in 70% of all patients with the highest frequencies in ATC (91%), PDTC (89%), and PTCFV (83%) patients. Pulmonary metastasis was present among 59% of patients with highest frequencies of among patients with ATC (91%) followed by those with PTCFV (73%), and PDTC (66.7%). Bone metastases were present in 32% of all patients with the highest frequencies in patients with FTC (65%), OTC (62%), and PTCFV (53%).

### 4.2. Molecular Mutation

Table 2 summarizes common mutations in the cohort with an overall frequency of equal to or higher than 5%. In patients with ATC, *TP53* (58%) was the most common genetic alteration followed by *BRAF* (42%), *TERT* (33%), and *RAS* (25%). Most patients with ATC had at least two mutations (92%). Patients with MTC exhibited the highest frequency of *RET* fusion alterations (54%) followed by *RAS* (27%) and *ATM* (8%). The frequencies of co-mutations were lowest in patients with MTC with 27% having two co-mutations, but no patients had more than two co-mutations. The most frequent mutation in patients with FTC was *RAS* (40%) followed by *TERT* (25%), *BRAF* (10%), *ATM* (10%), and *RET* fusion (5%). In addition, 55% of patients with FTC had two or more co-mutations, 15% had three or more, and 15% at least four mutations. In patients with PTCCV, the most frequent alteration was *BRAF* (62%) followed by *TERT* (32%), and *RET* fusion (10%). In patients with PDTC the most frequent mutations were *TERT* (40%) and *BRAF* (40%) with similar frequencies, followed by *TP53* (30%) and RAS (20%). Notably, 70% of patients with PDTC had two or more, 60% had three or more, and 20% had four or more co-mutations. In patients with PTCFV, the most frequent mutation was *RAS* (43%) followed by *TERT* (30%), *BRAF* (23%) and *ATM* (7%). Seventeen percent of patients with PTCFV did not have any detectable pathogenic mutation. In OTC, the most common genetic alteration was *BRAF* (25%) and *TERT* (25%) with similar frequencies, followed by *TP53* (12%) and *ATM* (12%). 

### 4.3. PD-L1 Expression and Progression Free Survival

**Figure 1:**
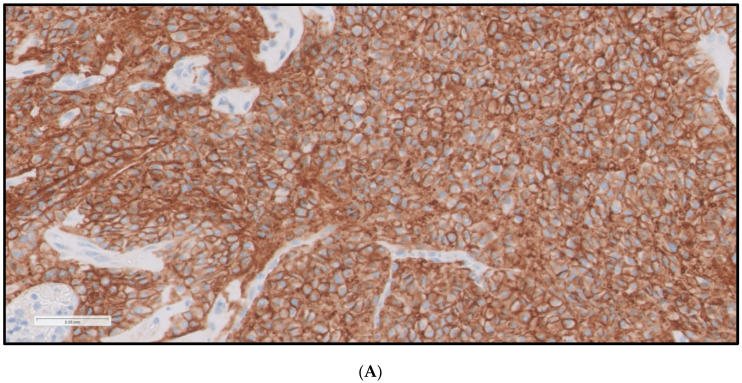

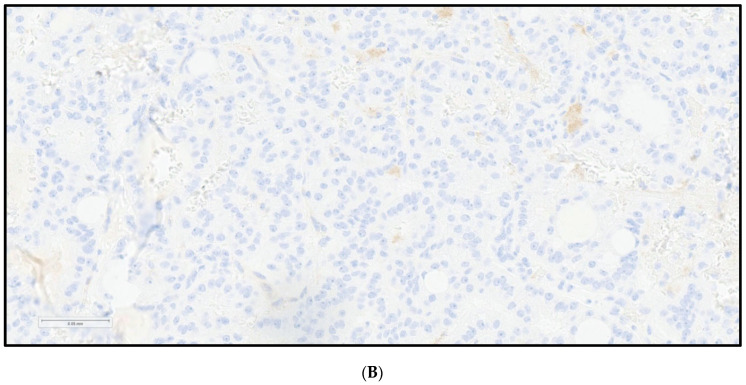
PD-L1 Staining. (**A**) Represents PD-L1 immunostaining (40× magnification) of TC from 63-year-old man with OTC. PD-L1 immunostaining was positive (strength 2+, 100% of cells examined—see methodology). (**B**) Represents PD-L1 immunostaining (40× magnification) of TC from 47-year-old women with PTCFV. PD-L1 immunostaining was negative in all cells examined.

Data for PD-L1 expression were available for 158 patients and were positive for 69% of patients with ATC, 71% of those with OTC, 11% of those with FTC, 10% of those with PDTC, and 28.5% of those with PTCCV. PD-L1 expression was not detected in patients with MTC and PTCFV (Figure 1). In our cohort, patients with MTC and PTCFV did not have detectable PD-L1 expression, and therefore, the analysis could not be performed in these groups. For other patient groups (FTC, PDTC, OTC), analysis of PFS between the PD-L1-positive and -negative groups could not be performed due to the small number of patients with PD-L1 expression. A subgroup analysis was performed for patients with ATC. 

**Figure 2:**
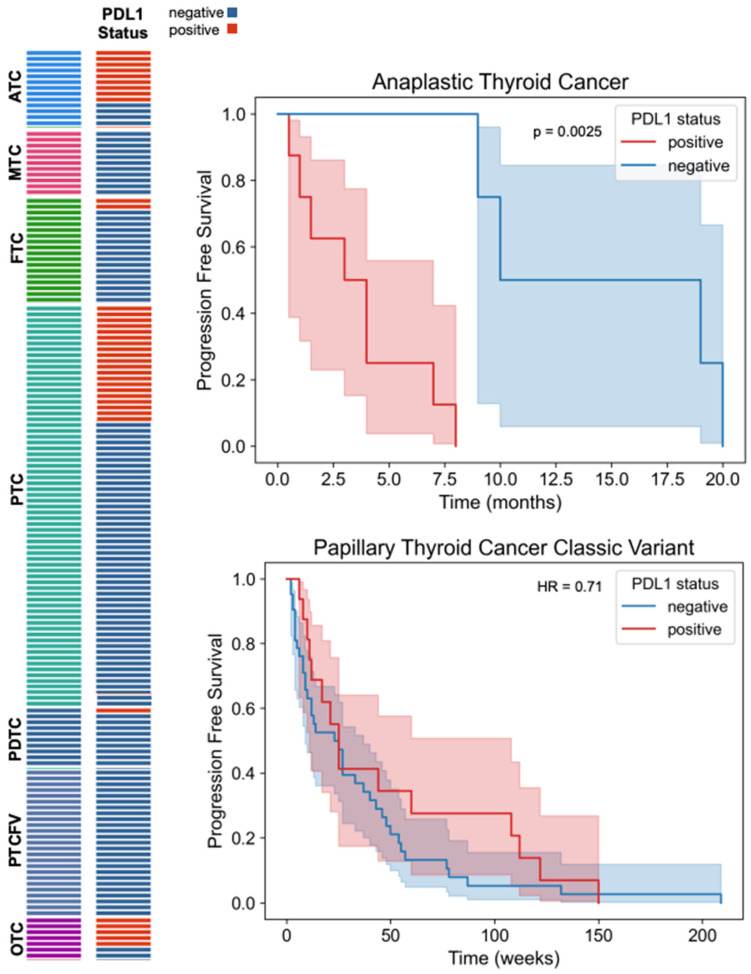
PD-L1 expression across different thyroid cancer types and histological subtypes and association with progression free survival (PFS) in ATC and PTCCV.

In ATC, patients with positive PD-L1 expression had a significantly lower progression free survival (PFS) compared to patients with negative PD-L1 expression (median survival time 3 months vs. 10 months; log-rank test *p* = 0.003). (Figure 2). In the ATC subset, patients with PD-L1-positive status and a BRAF mutation had a lower progression-free survival (PFS) compared to those who were PD-L1-positive without the BRAF mutation (log-rank test *p* = 0.01). A similar PFS was observed between PD-L1-positive patients with and without the TP53 mutation in the ATC subset. However, no significant interaction effects were observed between PD-L1 and either of the two mutations in the PTCCV subset. 

### 4.4. Association of PD-L1 Expression with Molecular Mutation

The frequency of *RAS* mutation was significantly different between patients with positive and negative PD-L1 expression: 8% in patients with positive and 24% with PD-L1 negative PD-L1 expression (*p* = 0.037). *TP53* mutation status was significantly higher in patients with PD-L1 expression: 22% in patients with positive and 7.5% with negative PD-L1 expression (*p* = 0.03) (Table 3). Patients with a *BRAF* mutation tended to have positive PD-L1 expression (*p* = 0.08). 

## 5. Discussion

Our study offers valuable insight into the variability of PD-L1 expression across different types and histological subtypes of TC, as well as its association with mutational status and clinical outcomes. Key findings include the identification of the heterogeneity of PD-L1 expression among TC types and histological subtypes, a significant link between PD-L1 expression and reduced PFS in patients with ATC, and a notable association between PD-L1 expression and specific genetic mutations. 

In our cohort, ATC tumors showed a high PD-L1 expression, with a frequency of 69%. Previous studies have reported PD-L1 expression in ATC with a frequency ranging between 22 and 65% [31,32,33,34]. Our findings align with the observation that ATC has a higher tumor mutation burden (TMB) compared to DTC and PDTC, suggesting greater genomic instability and accumulated genetic alterations in ATC [35,36,37]. The higher TMB observed in ATC may be underlying the observed higher expression of PD-L1. Tumor mutation burden, a measure of somatic mutation per mega base (Mb), serves as an indicator of tumor immunogenicity and the potential to produce high-quality neoantigens, thus enhancing T cell reactivity and responses to ICB therapies [38,39,40]. In contrast, MTC tumors in our cohort did not show any PD-L1 expression, despite reports of expression ranging from 6 to 25% in the literature [41,42,43]. This discrepancy may be due to our smaller cohort size, and variability in the sensitivities of the antibodies and threshold for positivity used in the literature. Notably, no PD-L1 positivity was observed in the PTCFV group. Although the clinical behavior and outcomes of PTCFV have been inconsistent [44,45,46], recent evidence suggest that PTCFV may represent an intermediate state between PTC and FTC based on its clinical phenotype and molecular landscape and likely also a distinct local immune response [18,47,48]. 

The high level of PD-L1 expression in ATC may indicate a more uniform immune response compared to the heterogeneous immune reaction observed in DTC. A high PD-L1 expression could also be constitutive. Future studies that also measure frequencies of CD8+ T cells could provide confirmation that the elevated PD-L1 observed is secondary to an acquired immune response. Evidence supports the variability in clinical presentation, disease progression and therapy responses among DTC subtypes and even within PTC [24,49]. The observed variability in PD-L1 expression among TC types and histological subtypes highlights the need for personalized biomarker profiling, integrating immune and molecular studies to tailor therapeutic strategies effectively. Information about molecular mutations could complement PD-L1 expression status in identifying appropriate immunotherapy candidates. Our study found that ATC tumors had the highest frequency of *TP53* mutations and PD-L1 expression. We identified a significant association between *TP53* mutation and PD-L1 expression. The *TP53* gene codes for a tumor suppressor involved in the regulation of the cell cycle and is the most frequently mutated gene in human cancer [50]. The *TP53* protein is considered to be a “gene guardian” due to its roles in inhibiting tumor occurrence and progression [51]. *TP53* mutation has been associated with increased PD-L1 expression in lung adenocarcinoma [52]. Further, *TP53* mutation can inhibit the innate immune signaling pathway and promote immune escape leading to decreased tumor infiltration of natural killer cells and T cells in a cancer specific basis [51,53,54,55]. The genetic landscape of ATC, including a higher frequency of *TP53* mutation may directly impact local tumor immunity, resulting in a more robust immune response compared to other TC types with lower *TP53* mutation frequencies [16,17,31,56,57,58].

Our study showed that PD-L1 expression was associated with a poor survival outcome in ATC. The PD-1/PD-L1 interaction inhibits T cell activation and proliferation, and cytokine production, leading to T cell exhaustion possibly contributing to the poor survival outcomes observed in PD-L1-positive patients. Immune checkpoint inhibitors (ICIs) that block this interaction have demonstrated efficacy in restoring T cell activation and enhancing T cell-mediated antitumor responses across various cancers [11,12]. Although immunotherapy has a shown limited efficacy in TC—about 6% in DTC with monotherapy or combined immunotherapies and 10–60% when addition of multi-kinase inhibitors—ATC has shown more promising results, with objective response rates (ORRs) ranging from 10 to 75%. Further studies are needed to explore the role of the mutational landscape, particularly *TP53* mutation, in influencing the local immune response in ATC and to determine if *TP53* mutation status can be used as an additional biomarker in combination with PD-L1 expression for selecting patients who may benefit from immune-based therapies in TC. 

## 6. Conclusions

Our study highlights the significant variability in PD-L1 expression across different TC types and histological subtypes, underscoring the importance of understanding tumor immunology and optimizing immunotherapeutic strategies in TC. The association between PD-L1 expression, mutational profiles, and PFS emphasizes the need for tailored therapeutic approaches in advanced TC. Further investigations into combining PD-L1 expression with genetic signature of the tumor, such as *TP53* mutation status, may improve patient selection for more personalized immunotherapy in advanced TC.

## 7. Limitations and Future Directions

Our study has several limitations. The retrospective design and potential for selection bias in the patient cohort may affect the generalizability of our findings. Additionally, the smaller sample sizes for certain TC types and subtypes reduces statistical power. Future research should involve larger cohorts to ensure sufficient power to detect significant differences across TC subtypes. The co-existence of thyroid autoimmune disease should also be confirmed in future studies and included in the analysis to further evaluate the significance of autoimmune diseases in TC local immune response. Further studies should also focus on elucidating the molecular mechanisms underlying the observed PD-L1 expression heterogeneity in TC, exploring its implication for immunotherapy.

## Figures and Tables

**Table 1:** Clinicopathological presentation of patients with aggressive thyroid cancer.

		Overall	ATC	MTC	FTC	PTCCV	PDTC	PTCFV	OTC
N		176	13	11	20	81	10	30	8
Gender (%)	Female	49	54	45	55	48	50	53	12
	Male	51	46	55	45	52	50	47	88
Age (Median [IQR])		59.0 [46.0, 69.5]	70.0 [66.0, 77.0]	62.0 [50.0, 67.0]	62.0 [45.8, 76.5]	54.0 [41.0, 66.0]	68.5 [64.2, 70.0]	60.5 [48.2, 67.0]	53.5 [46.8, 64.5]
Tumor Size Cm (median [IQR])		3.2 [1.7, 5.0]	4.6 [4.5, 6.8]	2.2 [1.5, 2.8]	4.3 [3.0, 5.0]	3.0 [1.5, 4.0]	7.0 [6.0, 7.5]	2.5 [1.7, 6.0]	5.8 [4.8, 7.0]
Involved Surgical Margins (%)		46.4	100	50	23.1	47.5	57.1	33.3	50
Extrathyroidal Extension (%)		43.4	100	55.6	21.4	45.3	71.4	21.7	n/a
Lymph Node metastasis in Central Compartment (%)		53	25	75	0	53	50	22	0
Distant Metastasis (%)		70.3	90.9	63.1	75	57.9	88.9	83.3	87.5
Pulmonary Metastasis (%)		59.3	90.9	27.3	60.0	52.6	66.7	73.3	50
Bone Metastasis (%)		32.4	11.1	27.3	65.0	15.8	33.3	53.3	62.5

ATC: Anaplastic Thyroid Cancer, MTC: Medullary Thyroid Cancer, FTC: Follicular Thyroid Cancer, PTCCV: Papillary Thyroid Cancer Classic Variant, PDTC: Poorly Differentiated Thyroid Cancer, PTCFV: Papillary Thyroid Cancer Follicular Variant, OTC: Oncocytic Thyroid Cancer.

**Table 2:** Molecular mutations (%).

	Overall	ATC	MTC	FTC	PTCCV	PDTC	PTCFV	OTC
N	176	13	11	20	81	10	30	8
*BRAF*	41.7	42	0	10	61.8	40	23.3	25
*RAS*	19.4	25	27.3	40	6.6	20	43.3	0
*TERT*	29.7	33.3	0	25	31.6	40	30	25
*TP53*	10.3	58.3	0	0	8	30	3.3	12.5
*RET*	8.6	0	54.5	5	10.5	0	0	0
*ATM*	5.7	0	9	10	5	0	7	12
No Known Pathogenic Mutations	8.6	8	0	15	5	10	17	0
**Co-Mutations**								
≥2 Mutations	58	92	27	55	58	70	50	50
≥3 Mutations	28	58	0	15	21	60	33	37
≥4 Mutations	10	10	0	15	7	20	7	0

ATC: Anaplastic Thyroid Cancer, MTC: Medullary Thyroid Cancer, FTC: Follicular Thyroid Cancer, PTCCV: Papillary Thyroid Cancer Classic Variant, PDTC: Poorly Differentiated Thyroid Cancer, PTCFV: Papillary Thyroid Cancer Follicular Variant, OTC: Oncocytic Thyroid Cancer.

**Table 3:** PD-L1 status and molecular mutation.

PD-L1 Expression				
Mutation	Overall	Negative	Positive	*p*-Value
N	157	120	37	
*BRAF*	68 (43.3)	47 (39.2)	21 (56.8)	0.08
*RAS*	32 (20.4)	29 (24.2)	3 (8.1)	0.03
*TERT*	48 (30.6)	38 (31.7)	10 (27.0)	0.7
*RET*	12 (7.6)	10 (8.3)	2 (5.4)	0.73
*TP53*	17 (10.8)	9 (7.5)	8 (21.6)	0.03

## Data Availability

Data is contained within the article.

## References

[1] Cabanillas M.E., McFadden D.G., Durante C. (2016). Thyroid cancer. Lancet.

[2] Lim H., Devesa S.S., Sosa J.A., Check D., Kitahara C.M. (2017). Trends in Thyroid Cancer Incidence and Mortality in the United States, 1974–2013. JAMA.

[3] Durante C., Haddy N., Baudin E., Leboulleux S., Hartl D., Travagli J.P., Caillou B., Ricard M., Lumbroso J.D., De Vathaire F. (2006). Long-term outcome of 444 patients with distant metastases from papillary and follicular thyroid carcinoma: Benefits and limits of radioiodine therapy. J. Clin. Endocrinol. Metab..

[4] Nixon I.J., Whitcher M.M., Palmer F.L., Tuttle R.M., Shaha A.R., Shah J.P., Patel S.G., Ganly I. (2012). The impact of distant metastases at presentation on prognosis in patients with differentiated carcinoma of the thyroid gland. Thyroid.

[5] Bible K.C., Kebebew E., Brierley J., Brito J.P., Cabanillas M.E., Clark T.J., Di Cristofano A., Foote R., Giordano T., Kasperbauer J. (2021). 2021 American Thyroid Association Guidelines for Management of Patients with Anaplastic Thyroid Cancer. Thyroid.

[6] Wells S.A., Asa S.L., Dralle H., Elisei R., Evans D.B., Gagel R.F., Lee N., Machens A., Moley J.F., Pacini F. (2015). Revised American Thyroid Association guidelines for the management of medullary thyroid carcinoma. Thyroid.

[7] Cabanillas M.E., Ryder M., Jimenez C. (2019). Targeted Therapy for Advanced Thyroid Cancer: Kinase Inhibitors and Beyond. Endocr. Rev..

[8] Fullmer T., Cabanillas M.E., Zafereo M. (2021). Novel Therapeutics in Radioactive Iodine-Resistant Thyroid Cancer. Front. Endocrinol..

[9] Ringel M.D. (2021). New Horizons: Emerging Therapies and Targets in Thyroid Cancer. J. Clin. Endocrinol. Metab..

[10] Zhang Y., Zheng J. (2020). Functions of Immune Checkpoint Molecules Beyond Immune Evasion. Adv. Exp. Med. Biol..

[11] Yamaguchi H., Hsu J.M., Yang W.H., Hung M.C. (2022). Mechanisms regulating PD-L1 expression in cancers and associated opportunities for novel small-molecule therapeutics. Nat. Rev. Clin. Oncol..

[12] Robert C. (2020). A decade of immune-checkpoint inhibitors in cancer therapy. Nat. Commun..

[13] (2018). Neoadjuvant PD-1 Blockade in Resectable Lung Cancer; Nivolumab and Ipilimumab in Advanced Melanoma; Overall Survival with Combined Nivolumab and Ipilimumab in Advanced Melanoma; Prolonged Survival in Stage III Melanoma with Ipilimumab Adjuvant Therapy; Combined Nivolumab and Ipilimumab or Monotherapy in Untreated Melanoma; Combined Nivolumab and Ipilimumab or Monotherapy in Untreated Melanoma; Nivolumab and Ipilimumab versus Ipilimumab in Untreated Melanoma; Rapid Eradication of a Bulky Melanoma Mass with One Dose of Immunotherapy; Genetic Basis for Clinical Response to CTLA-4 Blockade; Genetic Basis for Clinical Response to CTLA-4 Blockade in Melanoma; Nivolumab plus Ipilimumab in Advanced Melanoma; Safety and Tumor Responses with Lambrolizumab (Anti-PD-1) in Melanoma; Hepatotoxicity with Combination of Vemurafenib and Ipilimumab. N. Engl. J. Med..

[14] Reijers I.L.M., Menzies A.M., van Akkooi A.C.J., Versluis J.M., van den Heuvel N.M.J., Saw R.P.M., Pennington T.E., Kapiteijn E., van der Veldt A.A.M., Suijkerbuijk K.P.M. (2022). Personalized response-directed surgery and adjuvant therapy after neoadjuvant ipilimumab and nivolumab in high-risk stage III melanoma: The PRADO trial. Nat. Med..

[15] Forde P.M., Chaft J.E., Smith K.N., Anagnostou V., Cottrell T.R., Hellmann M.D., Zahurak M., Yang S.C., Jones D.R., Broderick S. (2018). Neoadjuvant PD-1 Blockade in Resectable Lung Cancer. N. Engl. J. Med..

[16] French J.D. (2020). Immunotherapy for advanced thyroid cancers—Rationale, current advances and future strategies. Nat. Rev. Endocrinol..

[17] French J.D., Bible K., Spitzweg C., Haugen B.R., Ryder M. (2017). Leveraging the immune system to treat advanced thyroid cancers. Lancet Diabetes Endocrinol..

[18] Cancer Genome Atlas Research N. (2014). Integrated genomic characterization of papillary thyroid carcinoma. Cell.

[19] Lawrence M.S., Stojanov P., Polak P., Kryukov G.V., Cibulskis K., Sivachenko A., Carter S.L., Stewart C., Mermel C.H., Roberts S.A. (2013). Mutational heterogeneity in cancer and the search for new cancer-associated genes. Nature.

[20] Xie Z., Li X., Lun Y., He Y., Wu S., Wang S., Sun J., He Y., Xin S., Zhang J. (2020). Papillary thyroid carcinoma with a high tumor mutation burden has a poor prognosis. Int. Immunopharmacol..

[21] Guo M., Chen Z., Li Y., Li S., Shen F., Gan X., Feng J., Cai W., Liu Q., Xu B. (2021). Tumor Mutation Burden Predicts Relapse in Papillary Thyroid Carcinoma with Changes in Genes and Immune Microenvironment. Front. Endocrinol..

[22] Alhejaily A.G., Alhuzim O., Alwelaie Y. (2023). Anaplastic thyroid cancer: Pathogenesis, prognostic factors and genetic landscape (Review). Mol. Clin. Oncol..

[23] Shobab L., Wartofsky L. (2023). Perspective: The Molecular Landscape of Radioactive Iodine Refractory Differentiated Thyroid Cancer and Poorly Differentiated Thyroid Cancer. Thyroid.

[24] Lu L., Wang J.R., Henderson Y.C., Bai S., Yang J., Hu M., Shiau C.K., Pan T., Yan Y., Tran T.M. (2023). Anaplastic transformation in thyroid cancer revealed by single-cell transcriptomics. J. Clin. Investig..

[25] Pu W., Shi X., Yu P., Zhang M., Liu Z., Tan L., Han P., Wang Y., Ji D., Gan H. (2021). Single-cell transcriptomic analysis of the tumor ecosystems underlying initiation and progression of papillary thyroid carcinoma. Nat. Commun..

[26] Chowdhury S., Veyhl J., Jessa F., Polyakova O., Alenzi A., MacMillan C., Ralhan R., Walfish P.G. (2016). Programmed death-ligand 1 overexpression is a prognostic marker for aggressive papillary thyroid cancer and its variants. Oncotarget.

[27] Shi R.L., Qu N., Luo T.X., Xiang J., Liao T., Sun G.H., Wang Y., Wang Y.L., Huang C.P., Ji Q.H. (2017). Programmed Death-Ligand 1 Expression in Papillary Thyroid Cancer and Its Correlation with Clinicopathologic Factors and Recurrence. Thyroid.

[28] Angell T.E., Lechner M.G., Jang J.K., Correa A.J., LoPresti J.S., Epstein A.L. (2014). BRAF V600E in papillary thyroid carcinoma is associated with increased programmed death ligand 1 expression and suppressive immune cell infiltration. Thyroid.

[29] Shaikh H., McGrath J.E., Hughes B., Xiu J., Brodskiy P., Sukari A., Darabi S., Ikpeazu C., Nabhan C., Korn W.M. (2021). Genomic and Molecular Profiling of Human Papillomavirus Associated Head and Neck Squamous Cell Carcinoma Treated with Immune Checkpoint Blockade Compared to Survival Outcomes. Cancers.

[30] Philip P., Azar I., Xiu J., Hall M., Hendifar A., Lou E., Hwang J., Gong J., Feldman R., Ellis M. (2022). Molecular Characterization of KRAS Wild-type Tumors in Patients with Pancreatic Adenocarcinoma. Clin. Cancer Res..

[31] Garcia-Alvarez A., Hernando J., Carmona-Alonso A., Capdevila J. (2022). What is the status of immunotherapy in thyroid neoplasms?. Front. Endocrinol..

[32] Zwaenepoel K., Jacobs J., De Meulenaere A., Silence K., Smits E., Siozopoulou V., Hauben E., Rolfo C., Rottey S., Pauwels P. (2017). CD70 and PD-L1 in anaplastic thyroid cancer—Promising targets for immunotherapy. Histopathology.

[33] Cameselle-Garcia S., Abdulkader-Sande S., Sanchez-Ares M., Rodriguez-Carnero G., Garcia-Gomez J., Gude-Sampedro F., Abdulkader-Nallib I., Cameselle-Teijeiro J.M. (2021). PD-L1 expression and immune cells in anaplastic carcinoma and poorly differentiated carcinoma of the human thyroid gland: A retrospective study. Oncol. Lett..

[34] Cantara S., Bertelli E., Occhini R., Regoli M., Brilli L., Pacini F., Castagna M.G., Toti P. (2019). Blockade of the programmed death ligand 1 (PD-L1) as potential therapy for anaplastic thyroid cancer. Endocrine.

[35] Landa I., Ibrahimpasic T., Boucai L., Sinha R., Knauf J.A., Shah R.H., Dogan S., Ricarte-Filho J.C., Krishnamoorthy G.P., Xu B. (2016). Genomic and transcriptomic hallmarks of poorly differentiated and anaplastic thyroid cancers. J. Clin. Investig..

[36] Pozdeyev N., Gay L.M., Sokol E.S., Hartmaier R., Deaver K.E., Davis S., French J.D., Borre P.V., LaBarbera D.V., Tan A.C. (2018). Genetic Analysis of 779 Advanced Differentiated and Anaplastic Thyroid Cancers. Clin. Cancer Res..

[37] Yoo S.K., Song Y.S., Lee E.K., Hwang J., Kim H.H., Jung G., Kim Y.A., Kim S.J., Cho S.W., Won J.K. (2019). Integrative analysis of genomic and transcriptomic characteristics associated with progression of aggressive thyroid cancer. Nat. Commun..

[38] Shao M.M., Xu Y.P., Zhang J.J., Mao M., Wang M.C. (2024). Tumor mutational burden as a predictive biomarker for non-small cell lung cancer treated with immune checkpoint inhibitors of PD-1/PD-L1. Clin. Transl. Oncol..

[39] Aggarwal C., Ben-Shachar R., Gao Y., Hyun S.W., Rivers Z., Epstein C., Kaneva K., Sangli C., Nimeiri H., Patel J. (2023). Assessment of Tumor Mutational Burden and Outcomes in Patients with Diverse Advanced Cancers Treated with Immunotherapy. JAMA Netw. Open.

[40] Efremova M., Finotello F., Rieder D., Trajanoski Z. (2017). Neoantigens Generated by Individual Mutations and Their Role in Cancer Immunity and Immunotherapy. Front. Immunol..

[41] Bi Y., Ren X., Bai X., Meng Y., Luo Y., Cao J., Zhang Y., Liang Z. (2019). PD-1/PD-L1 expressions in medullary thyroid carcinoma: Clinicopathologic and prognostic analysis of Chinese population. Eur. J. Surg. Oncol..

[42] Shi X., Li C.W., Tan L.C., Wen S.S., Liao T., Zhang Y., Chen T.Z., Ma B., Yu P.C., Lu Z.W. (2021). Immune Co-inhibitory Receptors PD-1, CTLA-4, TIM-3, LAG-3, and TIGIT in Medullary Thyroid Cancers: A Large Cohort Study. J. Clin. Endocrinol. Metab..

[43] Shi X., Yu P.C., Lei B.W., Li C.W., Zhang Y., Tan L.C., Shi R.L., Wang J., Ma B., Xu W.B. (2019). Association Between Programmed Death-Ligand 1 Expression and Clinicopathological Characteristics, Structural Recurrence, and Biochemical Recurrence/Persistent Disease in Medullary Thyroid Carcinoma. Thyroid.

[44] Carcangiu M.L., Zampi G., Pupi A., Castagnoli A., Rosai J. (1985). Papillary carcinoma of the thyroid. A clinicopathologic study of 241 cases treated at the University of Florence, Italy. Cancer.

[45] Chang H.Y., Lin J.D., Chou S.C., Chao T.C., Hsueh C. (2006). Clinical presentations and outcomes of surgical treatment of follicular variant of the papillary thyroid carcinomas. Jpn. J. Clin. Oncol..

[46] Hagag P., Hod N., Kummer E., Cohenpour M., Horne T., Weiss M. (2006). Follicular variant of papillary thyroid carcinoma: Clinical-pathological characterization and long-term follow-up. Cancer J..

[47] Yu X.M., Schneider D.F., Leverson G., Chen H., Sippel R.S. (2013). Follicular variant of papillary thyroid carcinoma is a unique clinical entity: A population-based study of 10,740 cases. Thyroid.

[48] Rivera M., Ricarte-Filho J., Knauf J., Shaha A., Tuttle M., Fagin J.A., Ghossein R.A. (2010). Molecular genotyping of papillary thyroid carcinoma follicular variant according to its histological subtypes (encapsulated vs infiltrative) reveals distinct BRAF and RAS mutation patterns. Mod. Pathol..

[49] Boucai L., Zafereo M., Cabanillas M.E. (2024). Thyroid Cancer: A Review. JAMA.

[50] Hainaut P., Pfeifer G.P. (2016). Somatic TP53 Mutations in the Era of Genome Sequencing. Cold Spring Harb. Perspect. Med..

[51] Yu J., Ling S., Hong J., Zhang L., Zhou W., Yin L., Xu S., Que Q., Wu Y., Zhan Q. (2023). TP53/mTORC1-mediated bidirectional regulation of PD-L1 modulates immune evasion in hepatocellular carcinoma. J. Immunother. Cancer.

[52] Dong Z.Y., Zhong W.Z., Zhang X.C., Su J., Xie Z., Liu S.Y., Tu H.Y., Chen H.J., Sun Y.L., Zhou Q. (2017). Potential Predictive Value of TP53 and KRAS Mutation Status for Response to PD-1 Blockade Immunotherapy in Lung Adenocarcinoma. Clin. Cancer Res..

[53] Ghosh M., Saha S., Bettke J., Nagar R., Parrales A., Iwakuma T., van der Velden A.W.M., Martinez L.A. (2021). Mutant p53 suppresses innate immune signaling to promote tumorigenesis. Cancer Cell.

[54] Lyu H., Li M., Jiang Z., Liu Z., Wang X. (2019). Correlate the TP53 Mutation and the HRAS Mutation with Immune Signatures in Head and Neck Squamous Cell Cancer. Comput. Struct. Biotechnol. J..

[55] Li L., Li M., Wang X. (2020). Cancer type-dependent correlations between TP53 mutations and antitumor immunity. DNA Repair.

[56] Menicali E., Guzzetti M., Morelli S., Moretti S., Puxeddu E. (2020). Immune Landscape of Thyroid Cancers: New Insights. Front. Endocrinol..

[57] Ma M., Lin B., Wang M., Liang X., Su L., Okose O., Lv W., Li J. (2020). Immunotherapy in anaplastic thyroid cancer. Am. J. Transl. Res..

[58] Gao X., Hong C., Xie Y., Zeng X. (2023). Immunotherapy or targeted therapy: What will be the future treatment for anaplastic thyroid carcinoma?. Front. Oncol..

